# Connexin43 contributes to electrotonic conduction across scar tissue in the intact heart

**DOI:** 10.1038/srep26744

**Published:** 2016-05-31

**Authors:** Vanessa M. Mahoney, Valeria Mezzano, Gary R. Mirams, Karen Maass, Zhen Li, Marina Cerrone, Carolina Vasquez, Aneesh Bapat, Mario Delmar, Gregory E. Morley

**Affiliations:** 1 Leon H. Charney Division of Cardiology, Department of Medicine, New York University School of Medicine, New York, NY,10016, USA; 2 Computational Biology, Department of Computer Science, University of Oxford, Oxford, United Kingdom

## Abstract

Studies have demonstrated non-myocytes, including fibroblasts, can electrically couple to myocytes in culture. However, evidence demonstrating current can passively spread across scar tissue in the intact heart remains elusive. We hypothesize electrotonic conduction occurs across non-myocyte gaps in the heart and is partly mediated by Connexin43 (Cx43). We investigated whether non-myocytes in ventricular scar tissue are electrically connected to surrounding myocardial tissue in wild type and fibroblast-specific protein-1 driven conditional Cx43 knock-out mice (Cx43fsp1KO). Electrical coupling between the scar and uninjured myocardium was demonstrated by injecting current into the myocardium and recording depolarization in the scar through optical mapping. Coupling was significantly reduced in Cx43fsp1KO hearts. Voltage signals were recorded using microelectrodes from control scars but no signals were obtained from Cx43fsp1KO hearts. Recordings showed significantly decreased amplitude, depolarized resting membrane potential, increased duration and reduced upstroke velocity compared to surrounding myocytes, suggesting that the non-excitable cells in the scar closely follow myocyte action potentials. These results were further validated by mathematical simulations. Optical mapping demonstrated that current delivered within the scar could induce activation of the surrounding myocardium. These data demonstrate non-myocytes in the scar are electrically coupled to myocytes, and coupling depends on Cx43 expression.

Cardiac injury, including myocardial infarction, leads to pathological tissue remodeling and fibrotic scarring. Replacement of myocytes with fibrotic tissue is associated with a significant risk of sudden cardiac death as a result of ventricular arrhythmias^1^,^2^. The mechanisms of arrhythmia formation in infarct scars and border zones remain incompletely understood. Studies of human^3^,^4^ and canine hearts^5^ have indicated that myocyte ion channel remodeling contributes to slow and heterogeneous conduction providing a substrate for reentry^6^,^7^,^8^. Although arrhythmic activity within the border zone region may in part be conducted by discrete, surviving intramural muscle bundles, the overwhelming number of non-myocytes that are also present may provide additional pathways for passive electrotonic conduction.

Cardiac scar tissue is composed of a phenotypically diverse group of non-myocyte cell types that include fibroblasts, myofibroblasts and a variety of neovascular cells. In addition to their important role in scar formation, there is growing evidence that fibroblasts play a direct role in the initiation and maintenance of arrhythmias through electrotonic coupling with myocytes and other fibroblasts. Although direct evidence in the intact heart is lacking, numerous *in vitro* experiments^9^,^10^,^11^,^12^,^13^,^14^,^15^ and clinical observations^11^,^16^,^17^,^18^ have highlighted the potential for coupling of fibroblasts and myocytes through gap junctions^15^. Cell culture and tissue experiments have indicated that the gap junction protein connexin43 (Cx43) is present in fibroblasts^12^,^14^,^19^,^20^ and studies in our laboratory have demonstrated that fibroblast Cx43 expression is enhanced following cardiac injury^21^.

Here, we provide the first direct evidence in the intact heart that the cells in myocardial scar tissue are electrically coupled to the surrounding myocardium and can support the passive conduction of action potentials. Using models of cardiac injury that produced transmural scars, we demonstrate that the cellular architecture of the scar tissue is consistent with a substrate capable of supporting electrotonic conduction. A series of distinct experimental approaches that included 1) optical mapping analyses, 2) direct microelectrode recordings and 3) *in silico* modeling established that the cells within a scar are capable of electrotonically conducting action potentials. Final validation of our findings was obtained by genetically targeting non-myocyte Cx43 expression and demonstrating that the gap junction protein is at least partially responsible for electrotonic conduction between myocytes and the cells in scar tissue.

## Methods

### Experimental Animals and Study Design

All procedures and protocols were approved by the Institutional Animal Care and Use Committee of the New York University School of Medicine, Protocol 130306-02, and complied with the standards for the care and use of animal subjects as stated in the *Guide for the Care and Use of Laboratory Animals.*

This study was performed using 2–4 month old male C57BL6 mice, and 2–4 month old male mice with fibroblast-specific protein 1 deletion of Cx43 (Cx43fsp1KO). Littermate Cx43^flox/flox^ Cre negative mice were used as controls for the studies that included Cx43fsp1KO mice. Cx43fsp1KO mice were generated as described in the Supplemental Material document.

### Cardiac Injury Models

Two injury models generating reproducible scars were used in this study.

#### Direct current-induced injury

A previously described direct current-induced injury model was used to study the electrophysiological interactions of myocytes and non-myocytes in relatively small scars in the right ventricle (RV)^22^. Briefly, mice were anesthetized with inhaled 1.5% isoflurane for the duration of the procedure. A subxiphoidal abdominal incision was made and a bipolar electrode (FHC Inc., Bowdoin, ME, USA) was advanced through the diaphragm using a micromanipulator and positioned in contact with the RV free wall near the apex. Pulsed direct current (8.0 mA, 100 ms interval, 30 ms pulse duration) was applied for 90 s to induce myocyte death. The electrode was removed, the incision was closed and the animal was allowed to recover for 30 days.

#### Left ventricular cryoinjury

Myocyte and non-myocyte interactions in larger injured regions were studied using a previously described cryoinjury model^23^. Mice were intubated and anesthetized with 1.5% isoflurane. A left thoracotomy at the 4^th^ intercostal space was performed to access the heart. A transmural cryoinjury was generated by placing a liquid-N_2_ chilled 3 mm circular flat-tip probe (Cry-Ac-3^®^, Brymill Cryogenic Systems) on the anterior left ventricular free wall surface for 10 seconds. The thoracotomy was closed, and the mice were allowed to recover for 30 days. Cryoinjured hearts were used to study the ability of non-myocyte regions to electrotonically conduct action potentials.

### Histology

Hearts were fixed with 4% paraformaldehyde in phosphate buffered saline (PBS), embedded in paraffin, and cut into 5 μm thick sections. Sections were stained with either Gomori’s trichrome or haematoxylin and eosin (H&E) according to the manufacturer’s instructions. Stained sections were scanned at a 40X magnification on a Leica SCN400F Whole Slide Scanner.

### Transmission Electron Microscopy (TEM)

Mice were anesthetized with 5% isoflurane and euthanized by cervical dislocation. Hearts were perfused with 15 ml PBS followed by 10 ml 4% paraformaldehyde. The RV free wall was excised and further fixed in 2.5% glutaraldehyde and 2% paraformaldehyde in 0.1 M phosphate buffer (pH 7.4) for 2 hours. After post-fixed with 1% osmium tetroxide for 1.5 hours, the samples were processed in a standard manner and embedded in EMbed 812 (Electron Microscopy Sciences, Hatfield, PA). Semi-thin sections were cut at 1 mm and stained with 1% toluidine blue to evaluate the quality of preservation. Ultrathin sections were cut at 60 nm, and stained with uranyl acetate and lead citrate by standard methods. Stained grids were examined under electron microscope (Philips CM-12) and photographed with a digital camera (Gatan; 4k x2.7k).

### Immunohistochemistry

Hearts were flash frozen in liquid nitrogen cooled isopentane and cryosectioned at 10 μm. Phalloidin (Alexa Fluor 594, Life Technologies, cat. A12381) and DAPI (Life Technologies, cat. D1306) stained sections were used to identify the scars. Sections were fixed in 100% acetone at −20 °C for 5 min then blocked for one hour in blocking solution containing (in mM) 20 Tris pH 7.4, 155 NaCl, 2 EGTA, 2 MgCl_2_ and 5% v/v Donkey Serum (Jackson Immunoresearch). Primary antibodies were incubated overnight at 4 °C or one hour at room temperature. Primary antibodies were diluted in blocking buffer as follows: Mouse anti-beta galactosidase (Promega, cat. Z3781) 1:100, chicken anti-Vimentin (Abcam, cat. 24525), rabbit anti-Cx43 (Millipore, cat. AB1727). Alexa conjugated secondary antibodies (Jackson Immunoresearch Laboratories) were used in a 1:200 dilution.

### Microelectrode recordings

Conventional glass microelectrodes (10–20 MΩ) were filled with 3 M KCl to record single cell transmembrane voltages in intact perfused hearts. The microelectrode recordings were obtained with a high input impedance amplifier (Electro 705 Electrometer, World Precision Instruments) equipped with capacitance neutralization. A reference electrode connected the bath to ground. Transmembrane potentials were low-pass filtered (cutoff 2 KHz) with Axon Instruments CyberAmp 380, digitized (5 KHz, sampling rate) with an Axon digidata A/D system and displayed with Axoscope 10.2 software. Analysis of recordings was performed using Clampex 10.4 software. Resting membrane potential (RMP), maximal signal amplitude, upstroke velocity (dV/dt), and signal duration at 70% repolarization (equivalent to action potential duration in myocytes) were evaluated. Measurements for each heart were obtained from 4–5 impalements in the center of the scar and uninjured remote regions.

### Optical mapping

Mice were heparinized (heparin sodium, 0.5 U/g IP) and euthanized by CO_2_ inhalation followed by cervical dislocation. Individual hearts were excised, the ascending aorta was cannulated, and the heart was Langendorff perfused with modified Tyrode’s solution as previously described^24^,^25^. For optical mapping studies, hearts were perfused with the excitation-contraction uncoupler blebbistatin (13.75 μM/L) and stained with the voltage-sensitive dye di-4-ANEPPS. Voltage dependent fluorescent signals were recorded using a modified microscope (MVX10 Olympus) equipped for epifluorescent illumination. Images were acquired with a 100 × 100 pixel array for 2 seconds at 1000 frames per second with a CMOS camera (SciMedia MiCAM ULTIMA). Continuous volume conducted electrocardiogram (ECG) recordings were obtained to monitor sinus rate. Hearts were excluded if sinus rate dropped below 240 beats per minute or if there was evidence of AV nodal block. Signal-to-noise ratio was calculated as the ratio of the fluorescent maximal signal amplitude and the root mean square of the optical noise recorded during diastole. Conduction velocity (CV) and signal duration at 70% repolarization were calculated from the optical signals as previously described^24^,^25^,^26^,^27^.

Direct electrical communication between myocyte and non-myocytes in the scar was assessed by evaluating the spread of voltage in space resulting from pulsed current delivered to a single area near the scar. Pulsed current (0.5–5.0 mA, at basic cycle length of 10 ms with 5 ms pulse duration) was delivered using a fire polished glass suction electrode (outer diameter of 1.2 mm) positioned approximately 1 mm from the border of the scar. Action potential generation was suppressed by adding 20 μg/ml of flecainide acetate (Almarytm, Meda Pharma) to the perfusate to allow for imaging of the spatial changes in passive membrane voltage in response to injected current. The optically recorded voltage deflection elicited by the pulsed current was measured 1–2 mm from the electrode and in the scar signal duration at 70% repolarization.

The electrical properties of the scar were assessed by pacing the RV free wall from several locations in the uninjured myocardium around the scar using bipolar electrodes (250 μm diameter, 800 μm separation; FHC Inc). The optical signal originating from the right side of the intraventricular septum was eliminated by positioning a piece of matte black aluminum foil in the RV cavity when necessary while performing optical mapping experiments with injured hearts. Signal amplitudes greater than twice background noise levels in the scar were considered to indicate the presence of electrotonic conduction.

S1 and S1–S2 stimulation protocols were used to study electronic conduction across the scar of cryoinjured hearts. The S1–S2 protocol consisted of ten S1 beats delivered at drive trains of 150–100 ms, followed by a single S2 beat delivered at progressively shorter intervals beginning at a coupling interval of 90 ms. The S2 delay was stepped down in 5 ms intervals until conduction was blocked in either the myocyte or scar pathways.

### Mathematical Modeling

Simulations were performed on two dimensional 5 × 5 mm tissue sheet using a previously described model for mouse ventricular myocytes^28^ with the introduction of a fibroblast membrane current model^29^ in a central 2 mm diameter circular region. Details of the mathematical simulations are included in the Supplemental Material document.

### Statistical Analysis

Results are presented as mean ± SEM. Student’s t-test was used to compare Cx43fsp1KO and controls for heart rate, body weight, and scar size. Student’s paired t-test was used for statistical comparison of voltage changes in response to pulsed current. Student’s paired t-tests were also used to compare electrophysiological parameters obtained from microelectrode recordings. Two factor ANOVA with replication and post hoc Student’s t-tests, when appropriate, were used to compare CV and APD values. Linear regression analysis was used to compare dV/dt values. A p-value less than 0.05 was considered to be statistically significant.

## Results

### Cx43fsp1KO mice

Cx43fsp1KO animals breed normally and offspring were generated at expected Mendelian frequencies. Heart rate and body weights of Cx43fsp1KO mice were not significantly different from littermate Cx43^flox/flox^ Cre negative controls (data not shown). In addition, ventricular CV and APD_70_ were not significantly different in Cx43fsp1KO mice compared with control animals (Supplemental Fig. 1).

### Scar Histology, TEM and Immunohistochemistry

**Figure 1:**
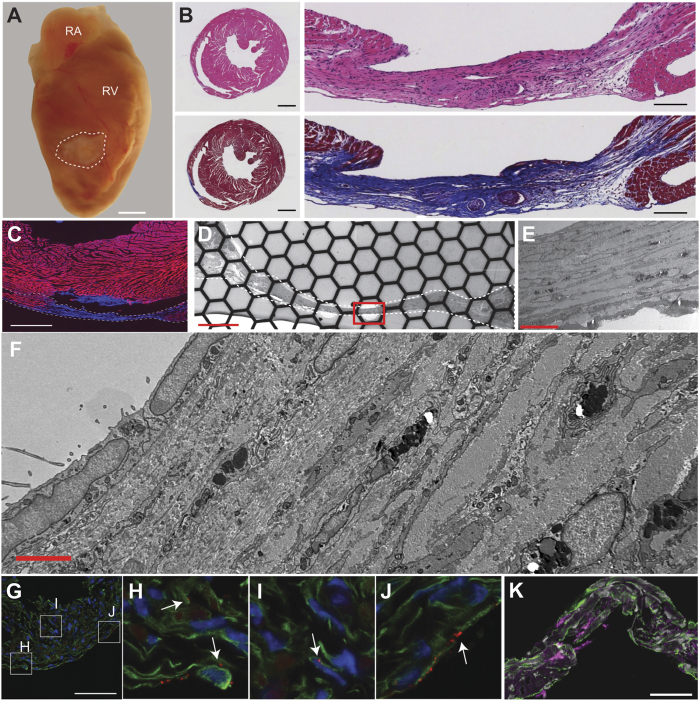
Histology and ultrastructure of ventricular scars. (**A**) Gross image of an injured heart (dotted line indicates scar border). RA, right atria; RV, right ventricle; bar = 1 mm. (**B**) Hematoxylin and Eosin and Masson’s Trichrome stained sections, top and bottom respectively. Cross section of heart at the level of the scar and magnified region showing scar, left and right respectively. Left bar = 1 mm, right bar = 100 μm. (**C**) Phalloidin (red) and DAPI (blue) staining of section including scar (dotted line), bar = 1.0 mm. (**D–F**) Transmission electron microscopy of a scar at different magnifications showing the cellular composition. (**E**) Enlargement of the area within the red box in D. (**F**) Enlargement of the area within the red box in E. Bar = 200, 10 and 2 μm, (**D–F**) respectively. (**G**) Immunohistochemistry of the scar shows abundant vimentin-positive cells (green), and punctate Cx43 signal (red). Bar = 50 μm. (**H–J**) Magnified image of boxes indicated in panel (**G**). (**K**) Section co-stained for vimentin (green) and β-galactosidase (magenta). Bar = 50 μm.

Histological analysis of the C57BL6 injured hearts was performed 30 days after the direct current injury procedure. The scar was identified macroscopically as a pale area on the right ventricular free wall (Fig. 1A). The mean scar surface area was 4.18 ± 0.287 mm^2^ (n = 24). H&E and Masson’s trichrome staining indicated the scars were densely packed with cells and contained significant collagen deposition (Fig. 1B). Phalloidin staining, which labels filamentous actin predominantly found in myocytes, was not present in the scars (Fig. 1C). Importantly, phalloidin staining of serially sectioned hearts demonstrated all of the injured hearts used in this study had transmural scars. The border between the uninjured myocardium and the scars was sharply demarcated and any surviving myocytes were within 50 μm from this border.

TEM indicated the scar was infiltrated by non-myocyte cells from epicardium to endocardium (Fig. 1D–F). The cells in the scar were small compared to myocytes and exhibited very thin (<100 nm width) extensions that met similar processes from other cells, suggestive of a cell network surrounded by extracellular matrix.

Consistent with other studies, immunohistochemistry revealed punctate Cx43 signal in the scar of C57BL6 injured hearts, and many Cx43 positive cells were also vimentin positive (Fig. 1G–J). To further confirm that Cx43 was expressed in non-myocyte cells in the scar, mice carrying Fsp1-Cre transgene were bred with Cx43 flox reporter mice (Cx43fsp1KO)^30^. Fsp1 is not expressed in cardiac myocytes^31^, therefore Cre recombinase is only present in non-myocyte cells. Upon Cre-mediated recombination, and deletion of Cx43, beta-galactosidase is expressed under the control of the endogenous Cx43 promoter. Beta-galactosidase expression was found in a subset of cells throughout the scars indicating that the Cx43 promoter region was active in non-myocyte cells (Fig. 1K). These data provide clear evidence that Cx43 is expressed in at least some of the cells within the scar. Scars in Cx43fsp1KO hearts were similar in size, histological, and gross appearance compared to littermate controls (Supplemental Fig. 2).

### Electrotonic coupling is reduced in the scar of Cx43fsp1KO hearts

**Figure 2:**
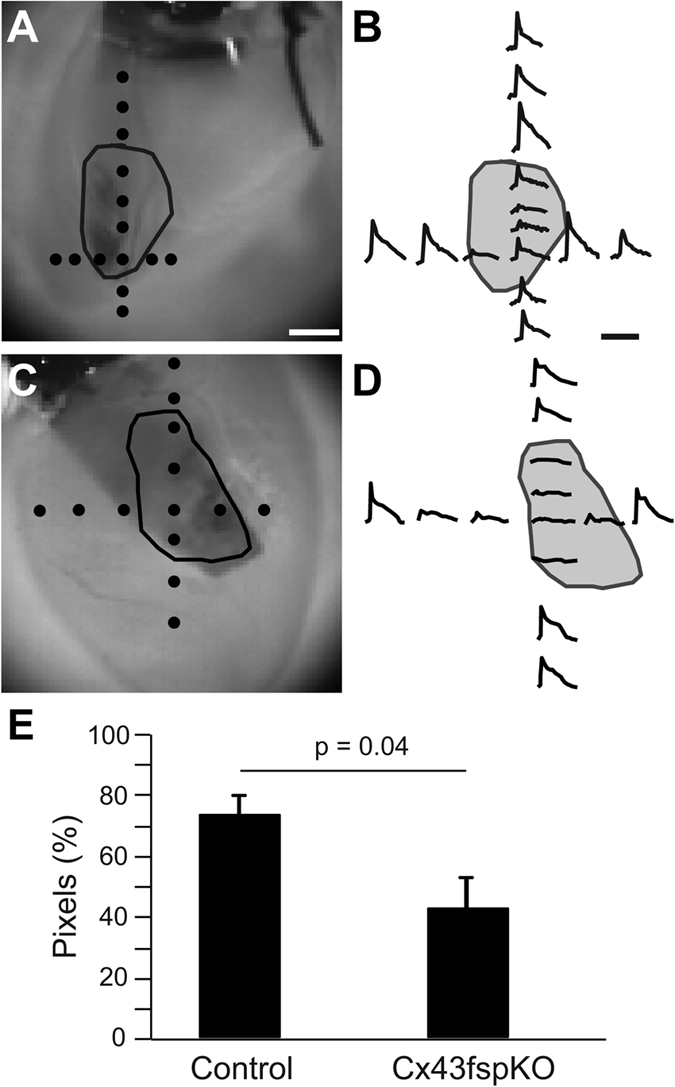
Electrotonic coupling between myocytes and non-myocytes in scars. (**A**,**C**) Brightfield images of RV free wall of a control and Cx43fsp1KO heart, respectively. Scar perimeter is indicated by the black line. Black dots indicate the location of the pixel traces shown on the right. Bar = 1 mm. (**B**,**D**) Individual pixel traces. Pixel amplitudes were normalized to the largest pixel amplitude on the RV free wall. Bar = 60 ms. (**E**) Chart shows the percentage of pixes within the scar region with signal-to-noise ratio greater than twice background levels. n =  6 and n = 7 for Control and Cx43fsp1KO, respectively.

The changes in membrane voltage in the scar during electrical activation of the RV free wall were evaluated with optical mapping (Fig. 2). A threshold of twice background noise levels was used to identify pixel locations showing the presence of electrotonically conducted signals. Pixels showing signal amplitude less than twice background noise levels were considered to be electrically silent. Using these criteria, analysis of single pixel traces across the RV free wall of control hearts demonstrated electrotonically conducted signals were present in more than 70% of the pixels in the scar (n = 6). Cx43fsp1KO mice were used to test if electrical communication between these cell populations is mediated by Cx43. Electrotonically conducted signals were also detected in the scar of Cx43fsp1KO hearts. However the percentage of pixels showing electrotonically conducted signals in the scar was significantly decreased compared with control hearts (n = 7). Together these data indicate cells in the scars are electrically coupled to the surrounding uninjured myocardium. Additionally, electrical coupling between these cell populations is least partly dependent on Cx43 expression in Fsp1 positive cells.

**Figure 3:**
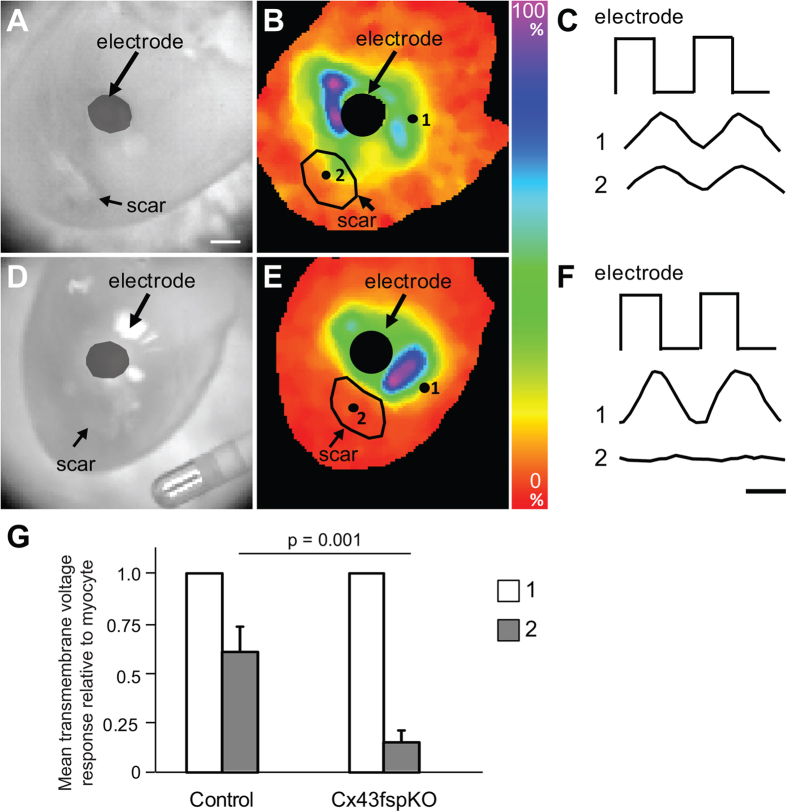
Electrotonic coupling is reduced in Cx43fsp1KO mice. (**A**,**D**) Brightfield images of RV free wall showing the location of the scar and electrode. Bar = 1mm. (**B**,**E**) Color map showing maximal voltage amplitude across the epicardial surface for a representative control and Cx43fsp1KO heart, respectively. Measurements were obtained at the peak response to the injected current pulse. Color bar = amplitude normalized to the largest amplitude pixel. (**C**,**F**) Voltage response recorded from the myocardium near electrode (1) and the scar (2) of control and Cx43fsp1KO hearts, respectively. (**G**) Mean relative transmembrane voltage response in the myocardium near the electrode and the scar, normalized to myocyte signal. n = 6 and n = 8 for Control and Cx43fsp1KO, respectively.

To further evaluate direct electrical communication between cells in the uninjured myocardium and the scar, voltage dependent changes were imaged while applying pulses of current through a suction electrode placed on the myocardium adjacent to the scar (Fig. 3 and Supplemental Video 1). Flecainide was used to suppress action potential generation in response to pulsed current injection. The representative maximal voltage amplitude map from the control group indicates a distance dependent decrease in the magnitude of the voltage change within the uninjured myocardium with respect to the current injection site. In-phase voltage changes were evident throughout the scar in all control hearts (Fig. 3C). The maximal signal amplitude in the scar was reduced to 61% of the signal amplitude from the myocardium near the electrode (Fig. 3G, n = 6 hearts). Detection of membrane voltage changes within the scar indicates direct electrical communication between the uninjured myocardium and the cells in the scar.

Significant differences in the electrophysiological properties of the scars were evident in Cx43fsp1KO hearts. Voltage changes in response to the applied current pulses were significantly less pronounced in the scars of Cx43fsp1KO hearts compared with the changes observed the scars of control hearts (Fig. 3G). See Supplemental Video 2 for voltage response during a complete cycle of pulsed current in the Cx43fsp1KO heart. The maximal signal amplitude from the scars of Cx43fsp1KO hearts was significantly decreased compared with the signal amplitude from the scars in control hearts. In Cx43fsp1KO hearts, the scar maximal signal amplitude was 12% of the signal amplitude near the electrode (n = 8 hearts). Together these data indicate that deletion of Cx43 from Fsp1 positive cells significantly reduces coupling between cells in the uninjured myocardium and the scar.

**Figure 4:**
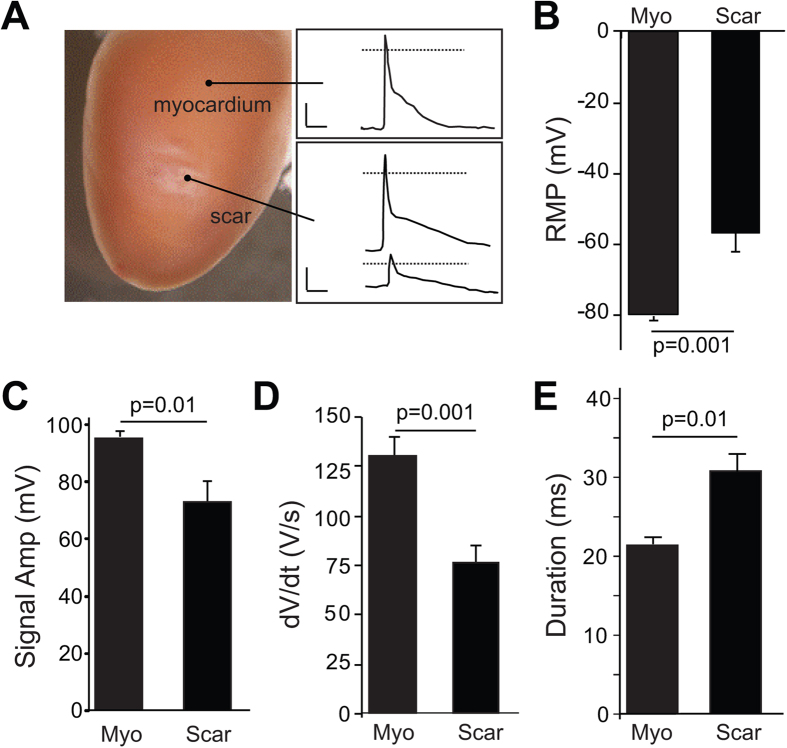
Microelectrode recordings from myocardium and scars. (**A**) Representative image (left panel) showing the two regions from which microelectrode recordings were obtained. Representative recordings obtained from the myocardium (right panel, upper box) and scar (lower box). Dotted line indicates 0 mV. Horizontal and vertical bars = 10 ms and 25 mV, respectively. (**B–E**) Average resting membrane potential (RMP), maximal signal amplitude, upstroke velocity (dV/dt), and signal duration at 70% repolarization, respectively. Data were obtained from n = 6 hearts. Average values were obtained for the myocardium and scar of each heart from a total of 4–18 impalements in each area.

Conventional microelectrode recordings were used to compare the characteristics of the electrical signals observed in the scar of control hearts with action potentials obtained from uninjured tissue (Fig. 4). The resting membrane potential of cells in the scar was significantly higher (−57.0 +/− 3.1 mV) than myocytes in the uninjured myocardium (−80.3 +/− 0.6 mV, n = 6 hearts). While some of the recordings from the scar closely followed myocyte action potentials (Fig. 4A, middle and top traces, respectively), there were also smaller amplitude signals observed throughout the scar (Fig. 4A, bottom trace). Figure 4B–E shows average RMP, maximal signal amplitude, dV/dt, and signal duration values at the center of the scar and uninjured remote regions, respectively (n = 6 hearts). Overall, maximal signal amplitude and dV/dt in the scar were significantly decreased compared to intact myocardium. In addition, the duration of the signals in the scar was significantly longer compared with the signals obtained from the myocardium remote from the scar. Since the cells in the scar are non-excitable, the presence of these signals in the scar indicates electrotonic coupling is present between the cells in the scar and uninjured regions of control hearts. In contrast, microelectrode recordings indicated electrical signals were absent in the scars of Cx43fsp1KO hearts (20 impalements from n = 5 hearts). Electrophysiological parameters including RMP, maximal signal amplitude, dV/dt and signal duration from uninjured myocardium of Cx43fsp1KO mice were not significantly different compared with control hearts (data not shown).

### Electrotonic conduction of action potentials

**Figure 5:**
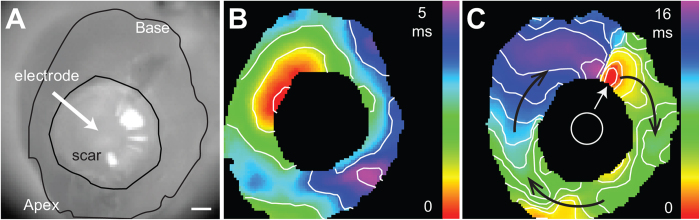
Current delivery within the scar can excite the surrounding myocardium. (**A**) Brightfield image of LV free wall showing placement of suction electrode and location of scar. Bar = 1 mm. (**B**) Activation map obtained during sinus rhythm. (**C**) Activation map obtained when the heart was paced with a suction electrode positioned within the scar (white circle). White arrow points to the site of earliest activation. Black arrows indicate the direction of propagation. Isochronal lines are drawn every 1 ms.

The LV cryoinjury model was used to determine if current delivered to the cells in the scar could electrically excite the surrounding myocardium. These experiments required larger scars that could accommodate attachment of a suction electrode for current injection. Similar to the right ventricular injury model, phalloidin and DAPI staining of serial sections was used to confirm cryoinjury produced transmural scars (data not shown). Figure 5 shows activation maps obtained during sinus rhythm and while injecting current at the center of the scar. Patterns of ventricular activation during sinus rhythm varied between hearts. In 5 out of 5 hearts, delivery of depolarizing current (0.5–1.0 mA pulse amplitude, 4 ms duration) within the scar was sufficient to excite and capture the surrounding myocardium at a 100 ms basic cycle length. Capture of the surrounding myocardium failed when suction was released while continuing to deliver current pulses, suggesting that pacing of the surrounding myocardium was not the result of field stimulation.

**Figure 6:**
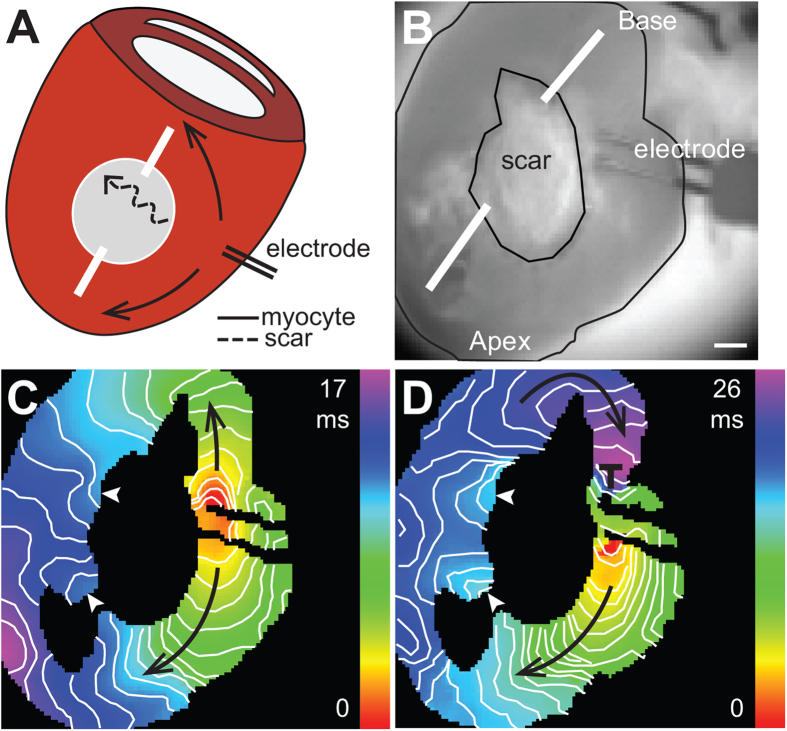
Electrotonic conduction across scars. (**A**) Schematic diagram of cryoinjury model showing transmural incisions and conduction pathways. Action potentials generated in the region proximal to the scar can be propagated to regions distal to the scar through active conduction through the myocyte pathway (myocyte), or by passive spread of current through the scar pathway (scar). (**B**) Brightfield image showing the location of the scar and pacing electrode. White lines show location of transmural incisions. Bar = 1 mm. (**C**,**D**) Activation maps of the S1 and S2 paced beats, respectively. S1 drive cycle was 100 ms; S2 was delivered 70 ms after the S1 beat. White arrowheads indicate the sites of earliest activation on the distal side of the scar. Black arrows indicate the direction of propagation. Region of conduction block is indicated by ┬. Isochronal lines are drawn every 1 ms.

Another series of studies (n = 6) were performed to determine if coupling within the scar would support passive electrotonic conduction of action potentials across the scar (Fig. 6 and Supplemental Videos 3–4). Action potentials generated in the region proximal to the scar can be propagated to regions distal to the scar through active conduction through myocytes (myocyte pathways) or by passive spread of current through the scar (scar pathway). Transmural incisions were created to structurally alter the myocyte pathway and facilitate electrical activation of the myocytes on the distal border of the scar through the scar pathway. The activation maps show discrete points of early activation on the distal border of the scar (n = 5), indicating electrotonic spread of current through the scar contributes to activation the myocardium at these sites (Fig. 6C). In a subset of hearts (n = 4), an S1–S2 pacing protocol was used to induce functional block and further delay activation through the myocyte pathway. Figure 6D shows a representative activation map of the S2 beat. Conduction slows through the lower myocyte pathway and block occurs near the electrode in the upper myocyte pathway. The changes in conduction properties of the myocyte pathways allows for the myocytes on the distal border of the scar to be fully activated by the current provided through the scar pathway. This observation is evidenced in the activation maps by the presence of concentric isochrones on the myocardium immediately distal to the scar. These findings demonstrate that the passive spread of current across non-myocyte gaps can contribute to electrical activation of the myocardium.

### Mathematical modeling of conduction in injured hearts

**Figure 7:**
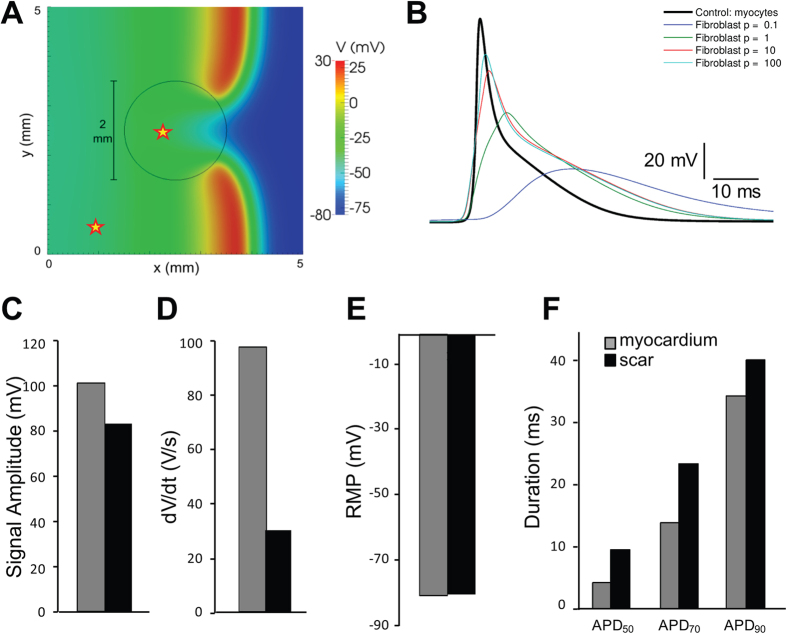
Simulation of conduction across non-myocyte scars. (**A**) Voltage map obtained with ρ = 1 showing the wavefront crossing the scar. The scar border is indicated by the black circle. The myocardium and scar measurement sites are shown with stars. (**B**) Scar transmembrane voltage obtained varying ρ between 0.1 and 100. (**C–F**) Maximal signal amplitude, upstroke velocity (dV/dt), resting membrane potential (RMP), and signal duration, respectively (ρ = 100).

Mathematical simulations of conduction across cardiac tissue containing a non-excitable scar were performed to determine if current mathematical models can predict the electrophysiological properties of the scar. Figure 7 shows a summary of the simulation data. The parameter C (which scales the ratio σ/χ, where σ represents cell to cell conductivity and χ is the density of membrane surface area) determines the properties of the electrotonic signals obtained from scar cells. See Supplemental Methods for a detailed explanation of how ρ influences passive electrical properties. Figure 7B shows the effects of varying ρ, between 0.1 and 100 in factors of 10, on the signals obtained from the scar. These data indicate that as the value of ρ increases, the simulated signals from the scar more closely resemble the microelectrode recordings shown in Fig. 4. Videos of these simulations are provided in the Online Supplement (Videos 5–8). The electrophysiological parameters in the myocardium and scar areas using ρ = 100 were qualitatively similar to the microelectrode data, i.e., signal amplitude and dV/dt were decreased in the scar, RMP was similar, and signal duration was prolonged in the scar (Fig. 7B–F). These data confirm that current mathematical models of cardiac conduction are broadly sufficient to explain the signals that were recorded in the injured regions.

## Discussion

Definitive proof of functional coupling between myocytes and non-myocytes in the heart has remained elusive since the concept was originally proposed in the 1960s^32^,^33^. Clinical findings^17^,^18^,^34^ and basic science studies^12^,^14^,^15^,^35^ have provided supporting evidence for electrical coupling between myocytes and fibroblasts in normal and diseased hearts. However, to date this fundamental question of cardiac electrophysiology has remained unanswered largely due to the inability to obtain convincing electrophysiological recordings from fibroblasts in the intact heart. This study aimed at providing definitive proof of electrical coupling between scar tissue and the surrounding uninjured myocardium. The results presented demonstrate passive spread of current across scar tissue can contribute to electrical activation of the myocardium. Further, coupling between myocytes and non-myocytes is mediated in part by Cx43.

Cardiac injury induces tissue damage that initiates an active process of scar formation. As the scar forms, the myocyte density in the area of injury further decreases partly mediated by apoptotic cell death^36^. At thirty days, a variety of cell types are present in the injured regions including, but not limited to, fibroblasts, myofibroblasts, macrophages and endothelial cells. Histological and ultrastructural analyses of the scars presented here are consistent with previous studies^6^,^37^,^38^,^39^ and demonstrate injured regions are richly populated with cells. Electron microscopy studies revealed a variety of cell types present in the scar. Serial sectioning and histological analyses confirmed myocytes were absent from large regions in the central portion of the scar. Additionally, fibroblast-like cells in the scars formed a mesh-like network surrounded by large spans of extracellular matrix. Although this arrangement of cells and matrix may provide mechanical integrity to the injured site, it also provides a substrate potentially capable of allowing for the passage of electrotonic current.

It has been suggested that coupling between myocytes and fibroblasts may be mediated by the gap junction proteins Cx40, Cx43 or Cx45, with prominent connexin isoform depending on the region of the heart^12^,^14^,^40^,^41^. Immunohistochemical analysis of connexin expression in scar regions following myocardial infarction have suggested fibroblasts express Cx43 and Cx45 in distinct temporal and spatial patterns^14^. Although these findings are important, due to the limitations of this technique, additional studies are needed to provide definitive evidence of fibroblast connexin expression and functional coupling in scar tissue. In this study, a novel genetic approach was used to target non-myocyte connexin expression by crossing Cx43 floxed mice with mice expressing Cre driven by the Fsp1 promoter. In this model, Cre-mediated recombination and deletion of Cx43 activated beta-galactosidase expression driven by the endogenous Cx43 promoter. The immunohistochemical data showing beta-galactosidase expression in the injured regions provided robust evidence that Cx43 is expressed throughout the scar. It is also evident from these data that targeted deletion of Cx43 occurred only in a subset of cells within the scar. As mentioned earlier, Fsp1 is not a specific fibroblast marker yet it is not expressed in cardiac myocytes, making it a very useful tool to target non-myocyte populations of cells in injured cardiac regions. Antibodies against Fsp1 protein recognize cells of myeloid origin in fibrotic liver and kidney^42^,^43^, and Fsp1 driven genetic labeling also identifies dendritic cells during pancreatitis^44^. The promoter construct that was used in this study has been shown to label CD11b+ myeloid cells in murine myocardial infarction models^45^, which would imply that a subset of the Cre+ cells that reside in the scar 30 days post ablation are myeloid or myeloid derived. Because of this issue we specifically refrain from labeling the scar cells as fibroblasts and further raise the possibility that other cells lineages, such as myeloid derived cells, might couple to cardiac myocytes in specific circumstances.

Evidence showing direct electrical coupling between myocytes and the cells within the injured tissue was obtained using optical mapping techniques. Direct current injection was used to establish passive spread of current from myocytes into the scar while blocking active membrane properties of the myocytes. Changes of membrane potential were observed throughout the scar indicating the presence of direct electrical connections between the uninjured myocardium and non-myocytes in the scar. Next optical mapping studies were performed to determine whether action potentials elicited in the myocardium were able to produce changes in membrane potential in the scar. Less than 30% of the pixels in the scar were considered to be electrically silent. The cells corresponding to these pixels were either weakly coupled or electrically uncoupled from the rest of the scar. In the remaining pixels (>70%), optical signals within the scar changed in phase with the surrounding ventricular myocardium. Consistent with passive spread of current, the amplitude of the signals within the scar were reduced relative to the surrounding myocardium. To further support this notion, the largest signal amplitudes were found near the edge of the scar while smaller signals were observed near the center of the scar.

Microelectrode recordings were used to characterize the electrophysiological properties of individual cells in the scar. The average resting membrane potential of the cells within the scar (−57.0 mV) was more depolarized compared to the surrounding myocardium (−80.3 mV). These values were consistent with previous measurements of resting membrane potential from cardiac fibroblasts and myofibroblasts which range from 0 to −70 mV^46^. The maximal amplitude and slope of the signals recorded from the scar was decreased compared with the action potentials recorded from the surrounding myocardium. These data are consistent with passive membrane depolarization of the cells within the scar by the surrounding myocardial tissue. Finally, the duration of the signals recorded from the scar was significantly longer than the surrounding myocytes. This is also consistent with the delayed passive discharge of cell membranes in the scar as the surrounding myocardium repolarizes. Although the optical mapping data from Cx43fsp1KO hearts indicated that voltage signals were present in some pixels in the scar, multiple attempts to obtain microelectrode recordings from the scars of Cx43fsp1KO hearts all failed. The apparent differences in these data sets are likely the result of the criteria used to identify the electrically silent regions in the optical mapping experiments, which led to false positive detection of electronically conducted signals in the scar. Together these data indicate that deletion of Cx43 from a subpopulation of cells in the scar is sufficient to disrupt electrical communication between the scar and the surrounding myocardium. At this point, we can only speculate about how deletion of Cx43 from the Fsp1 positive cells can disrupt electrical coupling within the scar. One possibility is that the cells form a network with a limited number of intercellular connections. If this is the case, disruption of electrical connections between a small number of cells could sufficiently modify the substrate in a way that impedes the spread of current throughout the scar. It is important to mention that these findings do not preclude the possible contribution of other connexin isoforms to intercellular coupling within the scar. Indeed, it is possible that deletion of other connexin isoforms could produce similar electrophysiological changes within the scar.

The larger cryoinjury model was used to demonstrate coupling within the scar could support passive electrotonic conduction of action potentials, and that the delivery of current to cells within the scar could excite the surrounding myocardium. Using this model we demonstrated direct current injection within the scar was sufficient to pace the heart. When suction was released, and current was allowed to pass in the extracellular space, capture was lost and normal sinus rhythm resumed. This again confirms electrotonic coupling between the cells in the scar and the surrounding myocardium. This also raises the interesting possibility that isolated surviving myocytes in infarct border zones could in fact act as a source for triggered electrical activity. To demonstrate the scar could support passive electrotonic conduction of action potentials, two incisions were used to facilitate electrical activation to travel across the scar. After the incisions were in place, conduction to the distal side of the scar could occur through the myocyte or scar pathways. Early electrical activation was detected originating from at least one site on the distal side of the scar indicating coupling within the scar could support passive conduction of action potentials. These findings are consistent with clinical studies demonstrating electrical conduction across transplant scars^17^,^34^, and *in vitro* studies in which electrical activation was able to passively conduct across fibroblast inserts^15^.

### Limitations

There are a few limitations of this study that should be mentioned. First, previous studies have demonstrated a variety of cell types actively contribute to the cardiac injury response. Although our studies demonstrated deletion of Cx43 in Fsp1 positive cells significantly reduces coupling in the scar, additional studies are needed to identify the specific cell types responsible for electrotonic coupling between the cells in the scars and myocytes. Given the number of cell types that may be involved, this is beyond the scope of the current study. Second, although the serial sectioning, immunohistological, and TEM approaches used did not identify a single myocyte that was more than 50 μm from the scar border, it is possible that a small number of surviving myocytes may have been present in some scars. The existence of a small number of isolated myocytes could facilitate passive conduction across scars. However, if any surviving myocytes were present within the scars, these myocytes would likely be isolated and would require to be coupled to other non-myocytes to electrically activate. Third, the injury techniques used in this study may generate an injury response that differs from response initiated by ischemic injury. The advantages of the injury models used in this study are that the location of the injury can be precisely controlled, and that the scars produced were fully transmural. Future studies are needed to confirm if electrotonic coupling between myocytes and non-myocytes occurs in other models of cardiac injury and disease. Fourth, Fsp1 has been shown to be expressed in a variety of cell types. Although the scar histology, size and structure were not altered in the Cx43fsp1KO hearts, we cannot exclude other functional changes including differences in proliferation, migration, or paracrine actions in these hearts. Finally, it is possible that current delivery with suction electrodes could have resulted in changes in the extracellular field, and these changes could contribute to the transmembrane voltages recorded within the scar. However, the reduced amplitude responses to injected current recorded in the Cx43fsp1KO scars strongly suggest the injected current is mainly traveling through the intracellular pathway.

## Additional Information

**How to cite this article**: Mahoney, V. M. *et al.* Connexin43 contributes to electrotonic conduction across scar tissue in the intact heart. *Sci. Rep.* **6**, 26744; doi: 10.1038/srep26744 (2016).

## Supplementary Material

Supplementary Information

Supplementary Video 1

Supplementary Video 2

Supplementary Video 3

Supplementary Video 4

Supplementary Video 5

Supplementary Video 6

Supplementary Video 7

Supplementary Video 8

Supplementary Video 9
