# Supplementary material for: Connexin43 contributes to electrotonic conduction across scar tissue in the intact heart
Source: Sci Rep. 2016 May 31;6:26744. doi: 10.1038/srep26744 (PMC4886689; doi:10.1038/srep26744)
Supplement: Supplementary Information [file srep26744-s1.pdf]

Connexin 43 contributes to electrotonic conduction across scar tissue in the intact heart

Vanessa M. Mahoney[1], Valeria Mezzano[1], Gary R. Mirams[2], Karen Maass[1], Zhen Li[1], Marina Cerrone[1], Carolina Vasquez[1], Aneesh Bapat[1], Mario Delmar[1], and Gregory E Morley[1]*

[1]Leon H. Charney Division of Cardiology, Department of Medicine, New York University School of Medicine, New York, NY,10016.

[2]Computational Biology, Department of Computer Science, University of Oxford, Oxford, United Kingdom.

SUPPLEMENTAL MATERIAL

EXPERIMENTAL ANIMALS

Cell-type specific connexin43 knockout mice (Cx43fsp1KO) were generated by crossing transgenic mice (BALB/c-Tg(S100a4-cre)1Egn/YunkJ) expressing Cre recombinase under the control of the mouse S100 calcium binding protein A4 (*Fsp1*) promoter [Bhowmick NA 2004] (Jackson Laboratories, stock #012641) to a Cx43 (*Gja1*) floxed mouse line (B6NCrl;129P2-Gja1tm1Kwi/Cnrm)(European Mutant Mouse Archive, EM:00327).[1] Each of these mouse strains were received as hemizygotes. Breeding was carried out to generate homozygous $Cx43^{flox/flox}$ mice, while only one copy of the Cre transgene was used in the mating pairs (noncarrier x hemizygote). Breeding pairs produce the expected Mendelian ratios, with 50% of the homozygous floxed Cx43 mice carrying the FSP1 Cre transgene. The colony is maintained by inbreeding of littermates. The mice used in this study have been backcrossed for at least 14 generations. Littermate $Cx43^{flox/flox}$ Cre negative mice were used as controls for the studies that included FSP1 Cx43 KO mice. Conduction system reporter mice (Cntn2-EGFP, CD1.Tg(Cntn2-EGFP)344sat) have been previously described[2] and are currently maintained in a CD1 background.

MATHEMATICAL SIMULATIONS

Simulations were performed using a previously described model for mouse ventricular myocytes.[3] The action potential model was modified by removing the non-inactivating steady state potassium current. This change increased action potential duration to more closely fit control experimental results.

The monodomain equation was used to simulate the electrical activity of a 2D square of tissue of dimensions 5mm x 5mm, with no-flux boundary conditions:

$$\chi\left(C_m\frac{\partial V}{\partial t}+I_{ion}\right)-\nabla.(\sigma\nabla V)=-I_{stim}$$

Here V is trans-membrane voltage, C_m is the capacitance of cell membrane (per unit area), χ is the membrane surface area per unit volume. Conductivity was set to be homogenous (no preferential fiber direction) with values of $\sigma = 1.75$ mS/cm along each axis. The action potential model that was used to calculate I_{ion} is the Li mouse ventricular model,[3] modified by removing the non-inactivating steady state potassium current to provide an increased action potential duration that was more in line with our control experimental results. The initial conditions for the Li model state variables were set to the

pseudo-steady values at 150 ms pacing cycle length (evaluated after 10,000 paces). I_{stim} is the stimulus current, which is applied along x = 0 at time t = 10.0 ms to allow the lesion region to settle to an equilibrium with the myocytes before the wavefront arrives.

The scar was modeled by introducing a circular region of 2 mm diameter in the center of the tissue where a fibroblast action potential model was used instead of the mouse ventricular myocyte model.[4] Inside the scar no stimulus current is applied, so we can simplify the equation above to be equal to zero. We can then divide through by χ to give:

$$C_m \frac{\partial V}{\partial t} + I_{ion} - \nabla.\left(\frac{\sigma}{\chi}\nabla V\right) = 0$$

This equation reveals that a parameter $p = \sigma/\chi$ governs tissue behavior, and this parameter can be altered to represent: (i) a change in density of membrane surface area in the tissue (χ); (ii) a change in the cell-to-cell coupling (σ); or (iii) both at once. Interestingly this shows, for instance, that a reduction to 10% conductivity together with 10% total cell membrane area gives $p = 0.1\sigma \div 0.1\chi = \sigma/\chi$, i.e. the behavior of the model would be unchanged. In other words, doubling the surface area of fibroblasts present in a given volume of the tissue has precisely the same effect as halving how well coupled they are, in terms of the evolution of the transmembrane voltage.

To simulate the initial injury we say that cell-cell coupling has been reduced dramatically (p=0.001). In this case the signals obtained from the lesion are substantially attenuated. We then simulate different levels of fibroblast coupling and cell density by varying ρ between 0.1 and 100 in factors of 10. Simulations were also performed to investigate the possible 3D source-sink effects of the reduced tissue thickness in the lesion (see Supplemental Video 9). Altering tissue thickness did not significantly differ compared with the 2D cases shown. The study was also performed with a 'neutral' action potential model in which I_{ion} is set to zero. The results for this simulation were again similar, suggesting the choice of fibroblast model does not strongly influence the findings of the model.

Numerical Implementation

Chaste[5] was used to solve the equations, using a finite element discretization with a space step of 0.005 cm and a PDE time step of 0.1 ms. The CVODE Backwards Differentiation Formulae solver[6] was used for solution of action potential model ODEs. Tthis solver takes adaptive time steps within the 0.1 ms PDE step to provide speed whilst meeting relative and absolute tolerances set to 10^{-5} and 10^{-7}

respectively. All of the code that was used is available open source as a bolt-on project for Chaste v3.2, available for download from http://www.cs.ox.ac.uk/chaste/download.html.

REFERENCES

1 Theis, M. *et al.* Endothelium-specific replacement of the connexin43 coding region by a lacZ reporter gene. *Genesis* **29**, 1-13, doi:Doi 10.1002/1526-968x(200101)29:1<1::Aid-Gene1000>3.0.Co;2-0 (2001).

2 Pallante, B. A. *et al.* Contactin-2 expression in the cardiac Purkinje fiber network. *Circ Arrhythm Electrophysiol* **3**, 186-194, doi:10.1161/CIRCEP.109.928820 (2010).

3 Li, L. *et al.* A mathematical model of the murine ventricular myocyte: a data-driven biophysically based approach applied to mice overexpressing the canine NCX isoform. *Am. J. Physiol. Heart Circ. Physiol.* **299**, H1045-1063, doi:10.1152/ajpheart.00219.2010 (2010).

4 Sachse, F. B., Moreno, A. P. & Abildskov, J. A. Electrophysiological modeling of fibroblasts and their interaction with myocytes. *Ann Biomed Eng* **36**, 41-56, doi:10.1007/s10439-007-9405-8 (2008).

5 Mirams, G. R. *et al.* Chaste: an open source C++ library for computational physiology and biology. *PLoS Comput Biol* **9**, e1002970, doi:10.1371/journal.pcbi.1002970 (2013).

6 Hindmarsh, A. C. *et al.* SUNDIALS: Suite of nonlinear and differential/algebraic equation solvers. *Acm T Math Software* **31**, 363-396, doi:Doi 10.1145/1089014.1089020 (2005).

LEGENDS FOR SUPPLEMENTAL VIDEO FILES

Supplemental Video 1. Optical mapping movie showing the voltage response during a complete cycle of pulsed current in an injured control heart. Flecainide was used to suppress action potential generation in response to pulsed current injection. The application of current produces changes in membrane potential in the surrounding myocardium and scar. Cold colors (blue, purple) represent the voltage at rest; bright colors (red, orange) show voltage response during current injection.This video corresponds to the voltage map shown in Figure 3B.

Supplemental Video 2. Optical mapping movie showing the voltage response during a complete cycle of pulsed current in an injured Cx43fsp1KO heart. Flecainide was used to suppress action potential generation in response to pulsed current injection. The application of current produces changes in membrane potential in the surrounding myocardium and scar. Cold colors (blue, purple) represent the

voltage at rest; bright colors (red, orange) show voltage response during current injection. This video corresponds to the voltage map shown in Figure 3E.

Supplemental Video 3. Optical mapping movie of an S1 paced beat (100 ms cycle interval) in a cryoinjured heart with transmural incisions. The corresponding activation map is shown in Figure 6C. The activation map shows discrete points of early activation on the distal border of the scar, indicating electrotonic spread of current through the scar contributes to activation the myocardium at these sites.

Supplemental Video 4. Optical mapping movie of an S2 paced beat at a coupling interval of 70 ms. Conduction slows through the lower myocyte pathway and block occurs near the electrode in the upper myocyte pathway. The changes in conduction properties of the myocyte pathways allows for the myocytes on the distal border of the scar to be fully activated by the current provided by through the scar pathway.

Supplemental Videos 5-8. Mathematical simulation of impulse propagation where scar ρ = 0.01, 1.0, 10 and 100, respectively. See Figure 7B for representative traces from lesion. Data from ρ =100 simulation was used to obtain the measurements in Figures 7C-F.

Supplemental Video 9. Mathematical simulation showing the 3D effect of electrical activity entering the thinner lesion tissue (ρ = 1). These data indicate the 3D effect was minimal for this value of ρ.

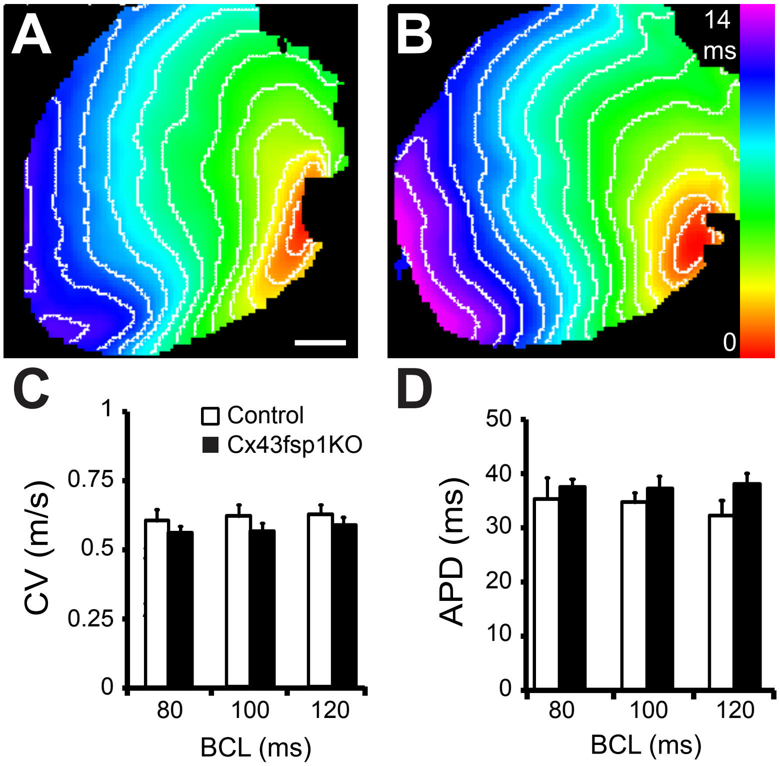

Supplemental Figure 1: Electrophysiological characterization of Cx43fsp1KO hearts.
A-B) Representative activation maps of the RV free wall obtained during epicardial pacing from a control and a Cx43fsp1KO heart, respectively. Scale bar = 1 mm. Isochronal lines are drawn every 1 ms. **C)** Average conduction velocity. p= 0.074 between control and Cx43fsp1KO; n = 9 per group. **D)** Average action potential duration. p = 0.563 between control and Cx43fsp1KO; n = 9 per group.

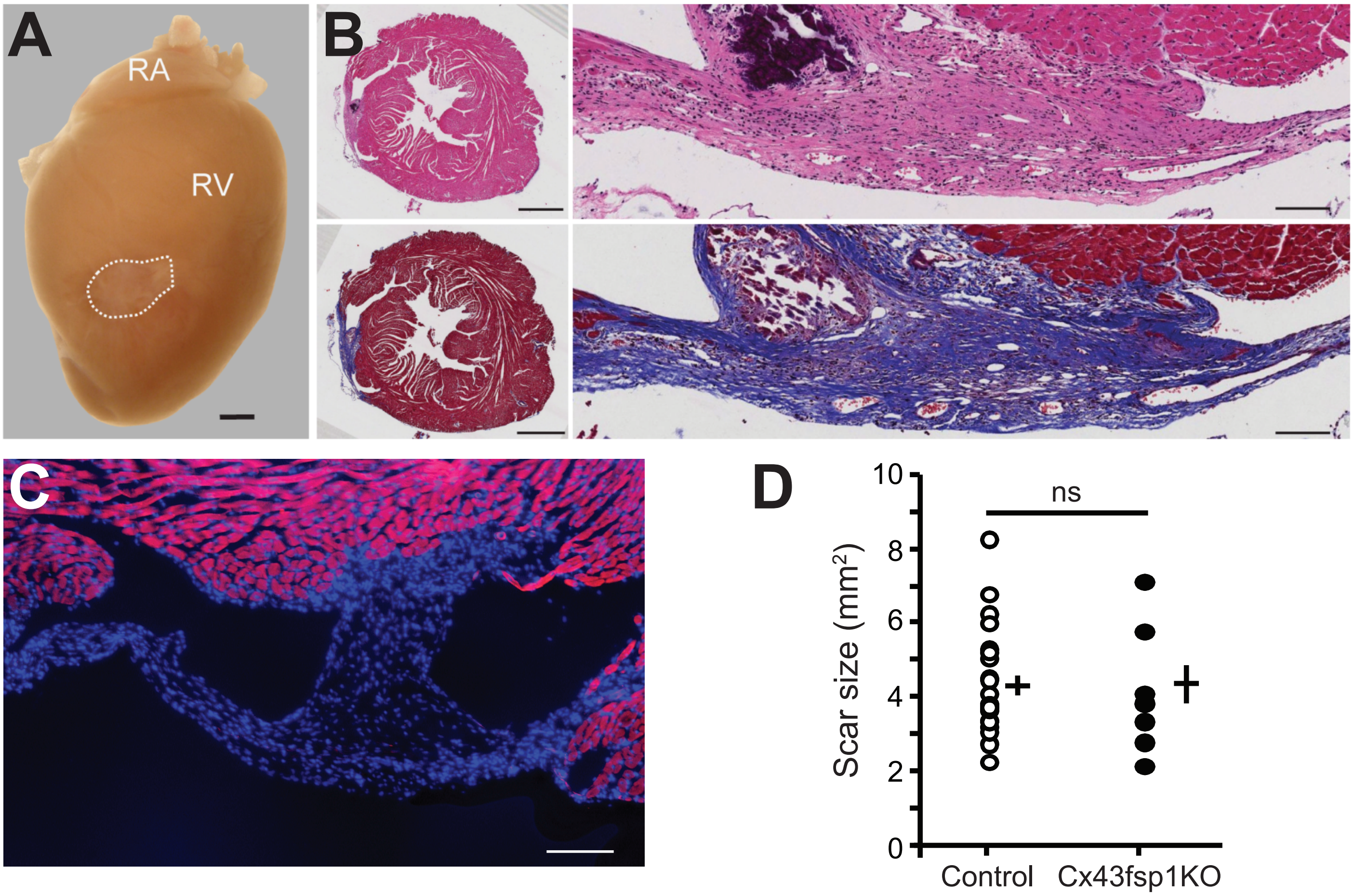

Supplemental Figure 2: Characterization of scars in Cx43fsp1KO hearts. A) Gross image of injured heart, dotted line indicates scar area. Bar = 1 mm. **B)** Hematoxylin and Eosin and Masson's Trichrome stained sections, top and bottom respectively. Cross section of heart at the level of the scar and magnified region showing scar, left and right respectively. Left bar = 1 mm, right bar = 100 µm. **C)** Phalloidin (red, myocytes) and DAPI (blue, nuclei) stained section. Bar = 100 µm. **D)** Scar area in control and Cx43fsp1KO mice. Symbols indicate individual scar measurements, horizontal lines indicate mean, vertical lines indicate SEM. ns, $p = 0.937$ (unpaired Student's t-test; $n = 24$ control, $n = 8$ Cx43fsp1KO).
